# The Tryptophan Biosynthesis Gene *trpE* Contributes to Virulence in Mesophilic *Aeromonas salmonicida* SRW-OG1

**DOI:** 10.3390/ani16162521

**Published:** 2026-08-12

**Authors:** Zhixing Zhang, Qiang Chen, Xiaojin Xu, Xufei Liu, Yujin Xiao, Chengcheng Li, Yuxuan Sha, Lunrui Zhang, Genhuang Xu, Guiling Zhang, Pocheng Chen

**Affiliations:** 1 Fisheries College, Jimei University, Xiamen 361021, China; 2 State Key Laboratory of Mariculture Breeding, Fujian Provincial Key Laboratory of Marine Fishery Resources and Eco-Environment, Fisheries College, Jimei University, Xiamen 361021, China

**Keywords:** *trpE*, *Aeromonas salmonicida*, Tryptophan biosynthetic, RNA-seq analysis, metabolomic analysis, *Epinephelus coioides*

## Abstract

The aquatic pathogen *Aeromonas salmonicida* SRW-OG1 is responsible for substantial mortality in *Epinephelus coioides* and many other cultured fish species. This study investigates the contribution of *trpE* to the pathogenicity of *A. salmonicida* SRW-OG1 in *E. coioides*. Specifically, *trpE* encodes a key enzyme in tryptophan biosynthesis; however, its function in regulating virulence in *A. salmonicida* remains uncertain. Phenotypic assays indicate that deletion of *trpE* markedly impaired bacterial growth and significantly reduced multiple virulence-associated traits, including swimming motility, biofilm formation, chemotaxis, hemolytic activity, and extracellular enzyme activity. Transmission electron microscopy revealed that the Δ*trpE* mutant lacked flagella, indicating that *trpE* is involved in flagellar formation or maintenance. In an *E. coioides* infection model, fish infected with Δ*trpE* showed a cumulative survival rate of 48.3%, whereas those infected with the wild-type SRW-OG1 suffered 100% mortality. The Δ*trpE* mutant also exhibited reduced bacterial loads in target organs (liver, head kidney, and spleen), while pretreatment with Δ*trpE* provided 53.3% protection against a subsequent wild-type challenge. Transcriptomic analysis identified 616 differentially expressed genes, mainly enriched in pathways including flagellar assembly, bacterial chemotaxis, two-component systems, quorum sensing, and biofilm formation. Metabolomic analysis further showed a significant decrease in N-butyryl-L-homoserine lactone, an acyl-homoserine lactone quorum-sensing signal in the Δ*trpE* mutant. These findings suggest that *trpE* contributes to mesophilic *A. salmonicida* SRW-OG1’s virulence by partially affecting quorum-sensing signal production and motility-related pathways. The attenuated phenotype and protective effect of Δ*trpE* indicate its potential as a live attenuated vaccine candidate for controlling *A. salmonicida* infection in aquaculture.

## Simple Summary

*Aeromonas salmonicida* causes severe mortality in grouper and other farmed fish, resulting in considerable economic losses in aquaculture. Although the *trpE* gene participates in tryptophan biosynthesis, its role in bacterial virulence remains unclear. Here, we investigated the function of *trpE* in *A. salmonicida* SRW-OG1. Deletion of *trpE* impaired bacterial growth, flagella formation and multiple virulence traits. In vivo assays confirmed that mutant exhibite attenuated virulence, and reduced tissue colonization, offering effective immune protection against wild-type infection. Omics analyses revealed that *trpE* modulates bacterial pathogenicity via quorum sensing and motility pathways, supporting its potential as a live attenuated vaccine candidate.

## 1. Introduction

*Aeromonas salmonicida* is a major aquatic pathogen responsible for fish furunculosis and bacterial septicemia. It is widely distributed in aquatic environments and infects a wide variety of host fish including *Salmo salar*, *Scophthalmus maximus*, *Cyprinus carpio*, and *Epinephelus coioides* the [1]. Infection via the oral cavity, gills, and bloodstream results in skin ulceration, muscle hemorrhage, abscess formation, and systemic septicemic symptoms [1]. As an important pathogen of both marine and freshwater fish, *A. salmonicida* can cause mortality rates of up to 90% in orange-spotted grouper (*E. coioides*), leading to economic losses accompanied by mortality in the aquaculture industry [2,3,4,5,6,7].

Tryptophan biosynthesis has been increasingly recognized as an important metabolic pathway closely associated with bacterial virulence [1,8]. As an aromatic amino acid, tryptophan influences the survival, colonization, proliferation, and pathogenicity within the host [8,9]. In addition, tryptophan-derived metabolites may participate in pathogen–host interactions and influence the expression of virulence-related factors [9,10]. Therefore, disruption of tryptophan biosynthesis may weaken bacterial fitness and reduce pathogenic potential [8,9]. The bacterial tryptophan biosynthetic pathway involves several enzymes, including TrpE, encoding the α-subunit of anthranilate synthase, crucial for the first committed stage of tryptophan biosynthesis, and TrpG [4]. More specifically, accumulating evidence from phylogenetically diverse microbial species indicates that the *trpE* gene is closely involved in microbial pathogenicity and virulence regulation, although its specific functional patterns vary significantly across taxa. In both pathogenic bacteria and fungi, the deletion or mutation of *trpE* impairs multiple virulence-related biological processes, including growth adaptability, biofilm formation, and pathogenic infection ability. Thus, *trpE* may be an important link between amino acid metabolism and bacterial virulence regulation.

Previous studies have suggested that mutation or inactivation of *trpE* may impair virulence-associated phenotypes in a range of microorganisms. Also, anthranilate, a key metabolite in the tryptophan synthesis pathway regulated by *trpE*, participates in the regulation of fungal developmental and pathogenic processes, inhibiting sexual mating and hyphal growth in *Sporisorium scitamineum*. In bacteria, *trpE* deficiency universally attenuates bacterial pathogenicity through multiple regulatory pathways. For instance, deletion of *trpE* significantly reduces biofilm formation and pathogenicity of *Ralstonia solanacearum* [11]. In *Ralstonia pseudosolanacearum*, the *trpE* mutation significantly reduces the expression of type III secretion system-related genes [8]. Similarly, deletion of *trpE* in *Salmonella enterica* serovar Typhimurium decreases bacterial adhesion and biofilm formation, whereas supplementation with exogenous tryptophan or indole can restore the biofilm-defective phenotype [12]. Such virulence-attenuating effects caused by impaired *trpE* function are not limited to specific bacterial groups. Similar results have been observed in diverse pathogenic microorganisms, including *Aspergillus fumigatus*, *Mycobacterium tuberculosis*, *Pseudomonas cannabina*, and *Xanthomonas citri* subsp. Citri [13]. Collectively, these cross-species studies suggest that *trpE* participates in the pathogenic regulation of diverse microorganisms, but its downstream regulatory pathways and phenotypic effects are species-specific rather than universally conserved. However, its function in *A. salmonicida* remains unclear.

To further investigate the pathogenic mechanism of this bacterium, a mesophilic *A. salmonicida* SRW-OG1 was isolated from a furunculosis symptomatic diseased orange-spotted grouper [14]. Unlike highly characteristic psychrophilic *A. salmonicida* strains, SRW-OG1 could grow normally at 37 °C, suggesting a high level of environmental adaptability and potential pathogenic risk [2]. A *trpE* deletion mutant, Δ*trpE*, and its complemented strain, C-Δ*trpE* isolated from *A. salmonicida* SRW-OG1, were constructed for this study. By comparing these strains with the wild-type SRW-OG1 strain, the effects of *trpE* deletion on bacterial growth, virulence-associated phenotypes, and pathogenicity were tested. Furthermore, transcriptomic and metabolomic analyses were performed to explore the molecular changes associated with *trpE* deletion and its linkage to bacterial virulence. By integrating phenotypic assays, infection experiments, and multi-omics analyses, this study aims to clarify the role of *trpE* in the virulence regulation of *A. salmonicida* to provide molecular evidence for its involvement in pathogenic processes.

## 2. Materials and Methods

### 2.1. Bacterial Strains, Corresponding Plasmids, and Growth Conditions

The *A. salmonicida* strain SRW-OG1, a mesophilic isolate recovered here from a diseased orange-spotted grouper from a marine farm in Zhangzhou, Fujian Province, China [2], served as the parental strain for this study. SRW-OG1 and its derivatives were routinely maintained on Luria–Bertani (LB) agar at 28 °C under static conditions, and cultivated in liquid LB broth at 28 °C with a shaking frequency of 220 rpm. *Escherichia coli* DH5α (Beijing Qushi Jin Biotechnology Co., Ltd., Beijing, China) was utilized as the host strain for plasmid construction and propagation, and cultured in liquid LB medium at 28 °C with shaking at 220 rpm. Chromosomal *trpE* knockout was generated using a modified λ-Red homologous recombination system adapted for *Aeromonas* species with slight revisions [4,10]. The helper plasmid pACYC184/Red encoding Exo, Bet and Gam recombinases (preserved in the laboratory) was transferred into competent *A. salmonicida* cells via electroporation to mediate homologous recombination. The pET-28a(+) vector (Shanghai Sangon Biotech Co., Ltd., Shanghai, China) was adopted to amplify homologous arms and resistance cassettes for preparing gene deletion fragments, while the suicide plasmid pJQ200SK (purchased from Shanghai Kerui Biotechnology Co., Ltd., Shanghai, China) was used to construct the complementary plasmid for generating the *trpE* complemented strain. The pACYC184/Red, pET-28a(+) and pJQ200SK plasmids were all stably preserved in *E. coli* cells, and separately cultured in LB broth supplemented with antibiotics at 28 °C and 200 rpm. Chloramphenicol was added to culture media to maintain the helper plasmid pACYC184/Red at (Cm, 25 μg/mL); kanamycin (Kan, 50 μg/mL) was applied to screen positive *trpE* deletion mutant colonies; gentamicin (Gen, 50 μg/mL) was used for the selection of the *trpE*-complemented strain; and ampicillin (Amp, 50 μg/mL) was used for routine identification of intermediate recombinant plasmids during vector construction.

### 2.2. Construction and Verification of Deletion and Complemented Strains

#### 2.2.1. Construction of the ΔtrpE Mutant

A Δ*trpE* deletion was created in SRW-OG1 by λ-Red-mediated homologous recombination. The pACYC184/Red plasmid was electroporated into SRW-OG1, and transformants were recovered on chloramphenicol-containing LB agar. A linear DNA fragment consisting solely of a kanamycin-resistance cassette flanked by 16 bp regions homologous to the sequences immediately upstream and downstream of *trpE* was then amplified from the pET-28a(+) template using primers *trpE*-F/*trpE*-R (Sangon Biotech) using 2× Accurate Taq Premix (Agbio Biotech, Hunan, China). The purified linear PCR fragment, excluding the entire pET-28a(+) plasmid backbone, served as the recombination substrate. Notably, conventional λ-Red requires long homologous arms, yet this modified λ-Red system, optimized for mesophilic *A. salmonicida* SRW-OG1, enables effective homologous recombination using short 16 bp homologous sequences [10]; this optimized strategy has been verified in previous gene knockout experiments targeting tryptophan metabolic genes in this strain. Following purification, the fragment was introduced by electroporation into SRW-OG1 carrying pACYC184/Red, and recombinants were selected on LB agar containing both kanamycin and chloramphenicol. Δ*trpE* mutants were first screened by colony PCR with primers *trpE*-p1/*trpE*-p2, and successful deletion was subsequently confirmed by Sanger sequencing together with a serial-passage assay to verify genetic stability.

#### 2.2.2. Construction of the Complemented Strain C-ΔtrpE

The complemented strain C-Δ*trpE* (pJQ200SK-*trpE*) was built using the suicide vector pJQ200SK (Kelei Biotechnology, Shanghai, China). A 1638 bp DNA fragment spanning the entire *trpE* coding sequence was PCR-amplified from wild-type SRW-OG1 genomic DNA with primers C-*trpE*-F/C-*trpE*-R, including the native *trpE* promoter. The pJQ200SK backbone was linearized by double digestion with SacI and XbaI (Takara Bio, Beijing, China), gel-purified with an EasyPure^®^ Fast Gel Extraction Kit (TransGen Biotech, Beijing, China), and ligated to the *trpE* amplicon using T4 DNA Ligase (Takara Bio, Beijing, China). The resulting construct, pJQ200SK-*trpE*, was heat-shock transformed into *E. coli* DH5α, and gentamicin-resistant transformants were verified by sequencing before the confirmed plasmid was electroporated into the Δ*trpE* mutant. The SRW-OG1 host strain supports pJQ200SK complementation plasmid’s episomal replication. Complemented colonies were selected on gentamicin-supplemented LB agar (50 μg/mL) and confirmed by PCR with primers *trpE*-p1/*trpE*-p2. All primers used for strain construction and verification are listed in Table 1.

### 2.3. Growth Curve Analysis

Growth curve assays of *A. salmonicida* SRW-OG1, Δ*trpE*, and C-Δ*trpE* were compared following a previously described method [15]. Overnight cultures of each strain were diluted in fresh LB broth to an OD_600_ of 0.1, then 20 μL of this bacterial suspension was added to 180 μL of LB broth per well of a 96-well plate. The plate was cultivated at 28 °C with orbital shaking at 220 rpm, and OD_600_ was recorded hourly over a 36 h period using a Synergy H1 microplate reader (BioTek, Winooski, VT, USA). Six biological replicates were included per strain.

### 2.4. Biofilm Formation Ability Assay

Biofilm-forming ability was quantified by crystal violet staining as described previously [16]. SRW-OG1, Δ*trpE*, and C-Δ*trpE* were each grown in LB broth at 28 °C, 220 rpm to mid-log phase, then standardized to 1.0 × 10^7^ CFU/mL based on OD_600_ values calibrated against colony counts. Two-hundred-microliter aliquots were dispensed into 96-well polystyrene plates; they were incubated statically for 16 h at 28 °C. After the wells were washed twice with PBS (pH 7.4) and air-dryed for 30 min, they were stained with 0.1% crystal violet (Merck KGaA, Darmstadt, Germany) for 20 min; unbound dye was removed by additional PBS washes. Bound crystal violet was solubilized in 200 μL of 33% acetic acid, and absorbance of that was read at 590 nm on a Synergy H1 microplate reader. Seven biological replicates were analyzed per strain, and differences among groups were assessed by one-way of variance (ANOVA) supplemented by Tukey’s post hoc test.

### 2.5. Swimming Motility Assay

Motility was assessed on 0.4% semi-solid LB agar as previously outlined [10]. Cultures of SRW-OG1, Δ*trpE*, and C-Δ*trpE* were grown to an OD_600_ of 0.5 and then adjusted to OD_600_ of 0.2 with fresh LB broth. A 1-μL aliquot of each strain was spotted at the center of a motility plate using a sterile pipette tip, and plates were incubated upright at 28 °C for 24 h. Migration diameters were measured with a JS-380A multifunctional colony counter (Shineso Biotech, Hangzhou, China). Three biological replicates were performed per strain, and each mutant was compared to wild-type SRW-OG1, and one-way ANOVA with Dunnett’s post hoc test was used for comparisons.

### 2.6. Chemotaxis Assay

Chemotactic behavior toward host mucus was evaluated using a modified capillary assay [17]. Wild-type SRW-OG1, Δ*trpE*, and C-Δ*trpE* strains were grown to mid-log phase in LB broth (28 °C, 220 rpm), washed twice in sterile PBS, and resuspended to OD_600_ of 0.3. A mucus-filled capillary tube (0.3 × 100 mm, sealed at one end) was inserted into a syringe holding 300 μL of the bacterial suspension and incubated at 28 °C for 2 h. The capillary contents were then expelled into 995 μL of PBS, serially diluted (10^−3^–10^−7^), and plated on LB agar for CFU enumeration. Sterile PBS served as the negative control, with three replicates per strain.

### 2.7. Hemolysis Assay

Hemolytic activity was measured using a modified plate assay [18]. Wild-type SRW-OG1, Δ*trpE*, and C-Δ*trpE* strains were grown to OD_600_ 0.5, then diluted to OD_600_ of 0.01 in fresh LB broth. Wells (6 mm diameter) were punched into sheep blood agar plates (HuanKai Microbial, Guangzhou, Guangdong, China) and loaded with 50 μL of the bacterial suspension. Following incubation at 28 °C for 24 h, the diameter of the resulting hemolytic halo around each well was measured with a JS-380A colony counter, and results were averaged across three independent experiments.

### 2.8. Extracellular Enzyme Activity Assays

Extracellular enzymatic activities were profiled by an agar well-diffusion approach [19]. Bacteria were grown to OD_600_ of 0.5 in LB broth, after which cell-free supernatants were prepared by centrifugation; following centrifugation at 12,000× *g* for 10 min at 4 °C, the supernatant was collected and filtered through 0.22-μm membranes. Caseinase, general protease, amylase, and lipase activities were tested on LB agar supplemented, respectively, with 0.4% casein, 2% skim milk powder, 1% soluble starch, or 1% Tween 80. Wells (6 mm) were loaded with 50 μL of filtered supernatant and plates incubated at 28 °C for 24 h. Hydrolytic zones were visualized with 10% trichloroacetic acid for the proteolytic assays, iodine staining for amylase activity, and direct precipitate visualization for lipase activity. Net zone diameters were recorded from three independent replicates per assay.

### 2.9. Transmission Electron Microscopy (Tem) Analysis of Bacterial Morphology

Cell and flagellar morphology were examined by TEM, wild SRW-OG1, Δ*trpE*, and C-Δ*trpE* were cultured in LB broth (28 °C, 220 rpm) for 12–18 h, adjusted to OD_600_ of 0.5, and two PBS washing steps were applied to the strains obtained after centrifugation at 8000× *g* for 2 min at 4 °C. A 10-μL droplet of each of the prepared suspensions was applied to a 300-mesh copper grid (Zhongjingkeyi, Beijing, China), and then adsorbing was carried out for 5 min; excess liquid was blotted by paper filtration, and grids were negative-stained using 2% phosphotungstic acid for 2 min before rinsing and air-drying. Samples were imaged with a Hitachi HT7800 transmission electron microscope (80 kV; Hitachi High-Tech, Tokyo, Japan) fitted with a Gatan Orius SC1000B camera (Gatan, Pleasanton, CA, USA). At least 20 cells were examined per strain.

### 2.10. In Vivo Infection Studies

#### 2.10.1. Experimental Fishes

Orange-spotted grouper (*E. coioides*, ~7.5 cm body length) collected from Zhangzhou, Fujian Province, were acclimated for 14 days in a recirculating aquaculture system at 28 ± 0.5 °C before challenge. Fish were fed once daily at 17:00 and fasted for 24 h prior to infection. The “Guide for the Care and Use of Laboratory Animals” served as the standard for all experimental operations of *E. coioides*, which obtained approval from the ethics committee of Jimei University (Approval No. JMULAC201159).

#### 2.10.2. Infection Experiments 

For the survival assay, a total of 240 *E. coioides* were separated into four treatment groups, with 60 fish each: a PBS control, wild-type SRW-OG1 infection, Δ*trpE* infection, and C-Δ*trpE* infection group. Fish in the bacterial infection groups were challenged intraperitoneally with 5 × 10^7^ CFU/fish of the corresponding bacterial strain, while the fish in the control group were injected with an equal volume of sterile PBS. The PBS group served as the negative control, and the SRW-OG1 group served as the positive infection control. Fish mortality was recorded daily.

A secondary lethal challenge assessed whether prior infection with the attenuated ∆*trpE* mutant could induce protective immunity against subsequent lethal wild-type bacterial reinfection using surviving individuals harvested from the primary survival trial. Two experimental subgroups were established (30 groupers per subgroup): a ∆*trpE*-pretreated group using fish from the ∆*trpE* primary infection group, and a PBS-pretreated control group using fish from the sterile PBS primary injection group. Fish from both subgroups were intraperitoneally challenged with 5 × 10^7^ CFU/fish of virulent wild-type SRW-OG1, and fish survival status was monitored daily for 10 consecutive days. No surviving fish from the wild-type SRW-OG1 primary infection group were enrolled in the secondary challenge, as nearly all individuals succumbed to acute infection during the first trial.

All survival statistical analyses were conducted in GraphPad Prism 9 (GraphPad Software, San Diego, CA, USA). Cumulative group survival curves were constructed using the Kaplan–Meier method. 

#### 2.10.3. Histopathological Examination and Tissue Sampling Collection

A separate cohort of 180 *E. coioides* was assigned to PBS, SRW-OG1, and Δ*trpE* infection groups for histopathological and bacterial-load analyses; fish in the infection groups were intraperitoneally injected with 5 × 10^7^ CFU/fish of the corresponding bacterial strain, whereas fish in the control group received sterile PBS.

At 4 days post-infection, three fish per group were euthanized; liver and head kidney tissues were collected for histopathological examination. Liver and head-kidney tissues were fixed in a paraformaldehyde–ethanol–acetic acid solution and shipped to Biomart Biological Co., Ltd. (Wuhan, China) for hematoxylin and eosin (H&E) staining. In parallel, liver, spleen, and head-kidney samples were snap-frozen in liquid nitrogen and stored at −80 °C for downstream bacterial quantification.

#### 2.10.4. Bacterial Load Quantification

Genomic DNA was extracted from liver, spleen, and head-kidney tissue using the EasyPure^®^ Marine Animal Genomic DNA Kit (TransGen Biotech, Guangzhou, China). Absolute quantification of bacterial load was achieved via the plasmid standard curve method. The full-length *gyrB* gene fragment of *A. salmonicida* SRW-OG1 was inserted into a pMD19-T plasmid to construct a standard plasmid. Purified standard plasmid was subjected to 10-fold serial dilution (10^1^–10^8^ copies/μL) to generate a linear standard curve for absolute copy calculation. The absolute *gyrB* copy number (*N*) of each tissue DNA sample was calculated by the formula: *N* = [*C* × 6 × 10^19^ ÷ (*M* × *X*)], where *C* = DNA concentration of a sample (ng/μL), *M* = average molecular weight of a single nucleotide, and *X* = total base length of the *gyrB* gene. The calculated *N* value directly represents the absolute bacterial burden in corresponding tissues. To facilitate inter-group comparison, relative bacterial load was normalized as the quotient of *gyrB* copy numbers in Δ*trpE*-infected samples divided by *gyrB* copy numbers in SRW-OG1 wild-type infected samples. Three technical replicates were arranged for each qPCR reaction to reduce experimental error. Bacterial burden was estimated by quantitative PCR targeting *gyrB* copy number, following an established protocol [20]. Primers for *gyrB* (Sangon Biotech; sequences in Table 2) were used with a QuantStudio 6 Flex Real-Time PCR System (Life Technologies, Carlsbad, CA, USA). The reaction was set up in a 10-μL volume, including 5 μL of Power SYBR Green PCR master mix, 0.25 μL each of forward and reverse primers, 0.5 μL of template DNA, and 4 μL nuclease-free water.

### 2.11. Transcriptomic (RNA-Seq) Analysis

Global transcriptional differences between SRW-OG1 and the Δ*trpE* mutant were characterized by RNA sequencing. Three independent biological replicates were prepared for wild-type SRW-OG1 and Δ*trpE* groups separately to ensure statistical reliability and experimental repeatability. Bacteria were harvested at logarithmic phase by centrifugation (5000× *g*, 30 min) and the supernatant was discarded. Total RNA was extracted from the resulting pellets using TRIzol reagent (Invitrogen, Carlsbad, CA, USA). RNA purity and integrity were assessed via NanoDrop spectrophotometry (OD_260_/_280_ = 1.8–2.2) and agarose gel was subjected to electrophoresis to screen qualified RNA for subsequent library construction. The ribosomal RNA was removed with the Ribo-Zero rRNA Removal Kit (Epicentre, Madison, WI, USA). Stranded libraries were built with the TruSeq™ Stranded Total RNA Library Prep Kit (Illumina, San Diego, CA, USA) before sequencing on an Illumina HiSeq 4000 platform (Guangzhou Kidio Biotechnology Co., Ltd., Guangzhou, China). Raw sequencing reads were quality-filtered by fastp to eliminate adapters, ambiguous N bases and low-quality reads; clean reads were mapped to the *A. salmonicida* SRW-OG1 reference genome using HISAT2, and gene expression abundance was quantified by RSEM. Differential expression was analyzed with DESeq2, and genes meeting *p* < 0.05 and |log_2_FC| > 1 were classified as differentially expressed (DEGs). Functional characterization of DEGs was carried out through Gene Ontology (GO; http://geneontology.org/, accessed on 24 July 2024) and KEGG pathway (https://www.genome.jp/kegg/, accessed on 24 July 2024) enrichment analyses [18,19]. The raw transcriptome data were analyzed using the DESeq2: v1.46.0 software. Genes that met the two conditions of *p* < 0.05 and |log: fold change (log_2_FC)| > 1 were defined as differentially expressed genes (DEGs). All the genes used for qRT-PCR verification were selected from this qualified DEGs set and were not included in those without significant differences or with low fold changes. We categorized differentially expressed genes into upregulated and downregulated groups, and selected them in a balanced manner: five significantly upregulated DEGs and five significantly downregulated DEGs were chosen to cover both opposing expression regulation patterns, thereby avoiding the bias that might result from focusing solely on one trend by quantitative reverse-transcription PCR (qRT-PCR) with primers detailed in Table 2.

### 2.12. LC-MS-Based Untargeted Metabolomic Analysis

Culture supernatants from wild-type SRW-OG1 and Δ*trpE* mutants were collected for untargeted LC-MS. Both strains were cultured in LB broth at 28 °C with shaking at 220 rpm until OD_600_ reached 0.5 ± 0.05; three independent biological replicates were set for each group. After centrifugation (4000× *g*, 15 min, 4 °C), supernatants were filtered through 0.22 μm sterile filters. For metabolite extraction, 400 μL of pre-cooled acetonitrile was added to 100 μL of filtrate for protein precipitation, followed by vortex mixing and incubation at −20 °C for 30 min. The mixture was centrifuged at 12,000× *g* for 15 min at 4 °C, and the supernatant was transferred to a new tube and vacuum-dried. Dried metabolites were reconstituted in 100 μL initial mobile phase solvent before LC-MS injection. QC samples were prepared by pooling equal volumes of all prepared samples to monitor instrument stability. All processed samples were stored at −80 °C pending instrumental detection.

The mobile phases consisted of 0.1% formic acid in water as phase A and 0.1% formic acid in acetonitrile as phase B. The gradient program was as follows: 0–2 min, 95–80% A; 2–5 min, 80–40% A; 5–6 min, 40–1% A; 6–7.5 min, 1% A; 7.5–7.6 min, 1–95% A; and 7.6–10 min, 95% A. Separation was performed at 40 °C with a flow rate of 0.4 mL/min and an injection volume of 4 μL.

Mass spectrometric detection was performed on a Q Exactive HF-X mass spectrometer (ESI± modes) equipped with dual-polarity ESI, operating in both positive and negative ion modes. The spray voltage was set at 3500 V in positive mode and 3200 V in negative mode, with sheath and auxiliary gases at 30 and 5 Arb, respectively. The vaporizer temperature was 300 °C, and the ion transfer tube temperature was 320 °C. The MS1/MS2 scan range was 75–1000 *m*/*z*, and data acquisition was performed using a resolution of 35,000 (MS1), an AGC target of 1 × 10^6^, and an RF lens value of 50%, followed by feature filtering (>50% missing values removed) using the K-nearest neighbor method. Peak areas were corrected using support vector regression. Metabolite annotation was performed using in-house, public, and prediction databases together with the metDNA algorithm. Only metabolites with an identification score ≥ 0.5 and a coefficient of variation (CV) < 0.3 in QC samples were retained for subsequent analysis. When metabolites were detected in both ion modes, the result with higher confidence and lower variation was prioritized. Quality control samples were taken every 10 samples to monitor instrument stability.

Multivariate analyses, including principal component analysis (PCA), hierarchical clustering, and orthogonal partial least squares discriminant analysis (OPLS-DA), evaluated statistically significant metabolic differences between groups. Differentially abundant metabolites were identified using the VIP > 1 and *p* < 0.05 thresholds. KEGG annotation and pathway enrichment analysis were conducted via a hypergeometric test, and a *p* < 0.05 was applied to identify significantly enriched pathways.

### 2.13. Statistical Analysis

All other statistical analyses were performed in SPSS 26.0 (IBM, Armonk, NY, USA), and data are expressed as mean ± standard deviation (SD). A one-way ANOVA compared multiple groups, and graphs were generated in GraphPad Prism 8.0.1 (GraphPad Software, USA) with statistical significance defined as *p* < 0.05. Tukey’s HSD post hoc test was applied for pairwise multiple comparisons following significant one-way ANOVA results across all phenotypic, bacterial load and histopathological datasets.

### 2.14. Data Availability

Table 1 primer pairs were designed according to the gene sequence (GenBank accession no. MT525253.1). Raw RNA-seq reads have been deposited in the GenBank^®^/Sequence Read Archive (SRA) database under accession number PRJNA1171403. The following accession numbers in the National Center for Biotechnology Information (NCBI) Sequence Read Archive (SRA) link to data used in this study: SRX26368371, CK_3; SRA: SRX26368372, E_1; SRA: SRX26368373, E_2; SRA: SRX26368374, E_3; SRA: SRX26368375. The CK group represents the wild-type SRW-OG1 strain, whereas the E group represents the *trpE* mutant. The expected target product sizes are 111 bp for *grcA* and *aspA*, 115 bp for *ompAI* and *flaA*, 112 bp for *napF* and *cysG*, 110 bp for *nirC* and *argF*, 113 bp for *cysD*, and 117 bp for *aerA*. Cumulative survival grouper survival curves were constructed according to the Kaplan–Meier method, with all statistical evaluations performed using GraphPad Prism 9 (GraphPad Software, San Diego, CA, USA). The metabolomics data of this study has been submitted to CNCB-NGDC: BioProject ID PRJCA070520, OMIX access number OMIX019144 (https://ngdc.cncb.ac.cn/omix/preview/6hlbImLk, accessed on 24 July 2024).

## 3. Results

### 3.1. Construction and Verification of the ΔtrpE Mutant and Complemented Strain

**Figure 1:**
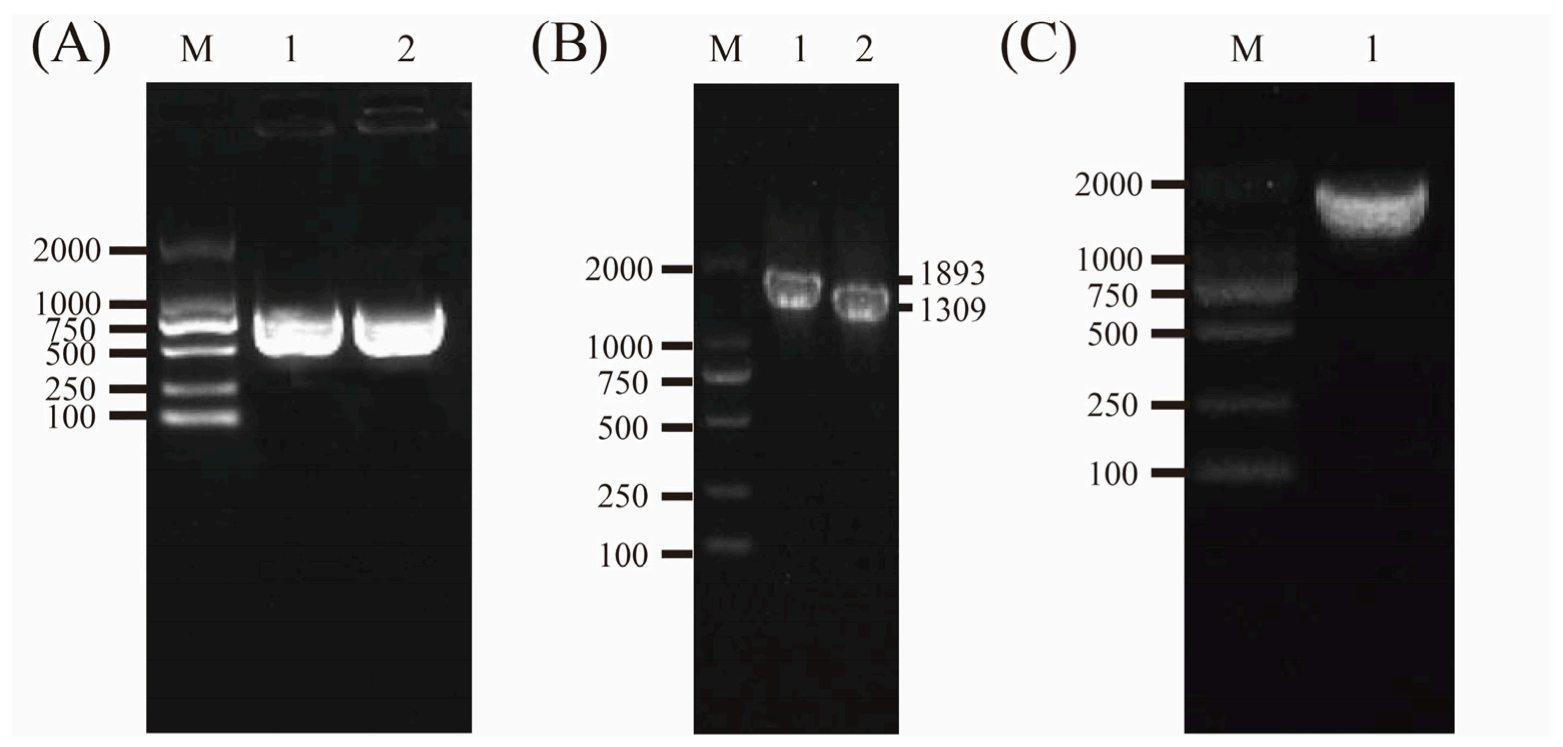
Confirmation of *trpE* mutant construction and complementation. (**A**) PCR amplification of the antibiotic resistance cassette used for allelic replacement; Lane M: DNA molecular weight marker; Lane 1, Lane 2: amplified target fragment (expected 845 bp). (**B**) PCR identification of wild-type strain and Δ*trpE* mutant; Lane M: DNA molecular weight marker; Lane 1: Wild-type SRW-OG1 (WT, band size = 1893 bp); Lane 2: Δ*trpE* mutant (band size = 1309 bp), verifying successful trpE gene knockout. (**C**) PCR verification of the C-Δ*trpE* complemented strain; Lane M: DNA molecular weight marker; Lane 1: C-Δ*trpE* complemented strain (band size = 1908 bp), confirming successful gene complementation. For all figure legends, WT indicates the wild-type *A. salmonicida* SRW-OG1 strain, Δ*trpE* indicates the *trpE* deletion mutant, and C-Δ*trpE* indicates the complemented strain.

To investigate the function of *trpE* in *A. salmonicida* SRW-OG1, a Δ*trpE* mutant was constructed using λ-Red homologous recombination. As shown in Figure 1, the kanamycin-resistant targeting fragment for *trpE* deletion was successfully amplified, with an expected size of 845 bp. PCR verification using primers *trpE*-p1/*trpE*-p2 confirmed that the amplified *trpE* fragment was 1893 bp in the wild-type SRW-OG1 strain, while the *trpE* gene fragment was replaced with a kanamycin fragment of a length of 1309 bp in the Δ*trpE* mutant strain. A garose gel electrophoresis, sequencing verification and continuous serial passage assays further confirmed the successful construction and genetic stability of the Δ*trpE* mutant.

The complemented strain C-Δ*trpE* was also successfully obtained by introducing the complete *trpE* gene into the Δ*trpE* mutant. PCR verification showed that the amplified *trpE* fragment in the complemented strain was 1908 bp, matching the expected size of the complemented gene (Figure 1C). Sequencing and multi-generation stability analysis confirmed the correct integration and stable genetic inheritance of the complemented *trpE* gene.

### 3.2. Deletion of trpE Impaired Virulence-Associated Phenotypes

To determine whether *trpE* contributes to the physiological fitness and virulence-related traits of *A. salmonicida*, the growth and several pathogenicity-associated phenotypes of wild-type SRW-OG1, Δ*trpE*, and C-Δ*trpE* were compared.

**Figure 2:**
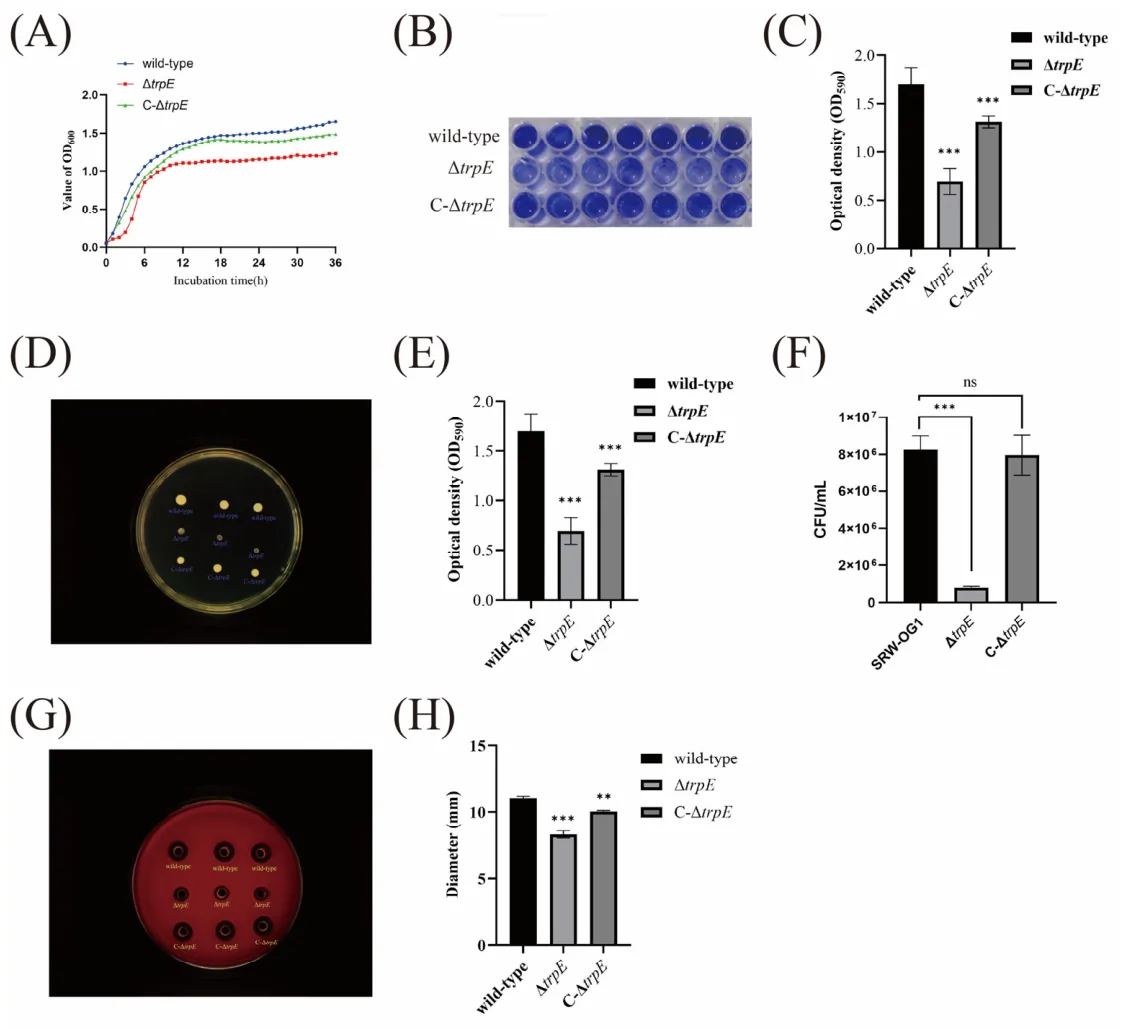
Comparison of growth, biofilm formation, motility, chemotaxis and hemolytic activity among wild-type SRW-OG1, Δ*trpE* mutant and *C-*Δ*trpE* complementary strains. (**A**) Comparison of the growth of the wild-type SRW-OG1, Δ*trpE* mutant strain, and C-Δ*trpE* complementary strain; (**B**,**C**) determination of biofilm formation ability of the wild-type strain, Δ*trpE* mutant strain and C-Δ*trpE* complementary strain; (**D**,**E**) comparison of colony diameters of the wild-type strain, Δ*trpE* mutant strain, and C-Δ*trpE* complementary strain on LB semisolid plates; (**F**) determination of chemotaxis ability of SRW-OG1 wild-type strain, Δ*trpE* mutant strain, and C-Δ*trpE* complementary strain; (**G**,**H**) hemolytic activity of WT, Δ*trpE*, and C-Δ*trpE,* determined on blood agar. ** *p <* 0.01, *** *p <* 0.001.

Growth curve analysis of SRW-OG1, Δ*trpE*, and C-Δ*trpE* strains showed that deletion of *trpE* impaired bacterial growth under the tested culture conditions. Compared with the wild-type SRW-OG1 strain, the Δ*trpE* mutant exhibited reduced growth, whereas growth was partially restored in the complemented strain C-Δ*trpE* (Figure 2A). This result indicates that *trpE* is associated with the normal growth capacity of SRW-OG1.

Biofilm formation was then evaluated using crystal violet staining. The wild-type SRW-OG1 strain showed strong biofilm formation, with an OD_590_ value of 1.657 ± 0.158. In contrast, the OD_590_ value of the Δ*trpE* mutant decreased to 0.696 ± 0.133 and was significantly reduced by 58% (*p <* 0.001) in biofilm biomass compared with SRW-OG1. The complemented strain C-Δ*trpE* showed partial recovery of biofilm formation, with an OD_590_ value of 1.313 ± 0.061, although the level remained lower than that of the wild-type SRW-OG1, with a 21% reduction relative to the SRW-OG1 strain, and this difference was also highly significant (*p <* 0.001) (Figure 2B,C).

Bacterial swimming motility was also markedly affected by *trpE* deletion. On LB semi-solid agar plates, the migration diameter of wild-type SRW-OG1 was 5.46 ± 0.25 mm, whereas that of the Δ*trpE* mutant decreased to 2.51 ± 0.25 mm (*p* < 0.001). Motility was partially restored in C-Δ*trpE*, with a migration diameter of 4.76 ± 0.33 mm, although this value remained significantly different from that of wild-type SRW-OG1 (*p* < 0.05) (Figure 2D,E).

Consistent with the reduction in swimming motility, bacterial chemotactic ability was strongly impaired in the Δ*trpE* mutant. The wild-type SRW-OG1 strain reached 8.26 × 10^6^ CFU/mL in the chemotaxis assay, whereas the Δ*trpE* mutant decreased to 7.9 × 10^5^ CFU/mL, corresponding to a 90.4% reduction (*p* < 0.001). In comparison, the complemented strain C-Δ*trpE* reached 7.96 × 10^6^ CFU/mL, showing no significant difference from the wild-type SRW-OG1 (*p* > 0.05) (Figure 2F).

Hemolytic activity was further assessed using blood agar plates. The wild-type SRW-OG1 strain produced a hemolytic zone of 11.09 ± 0.23 mm. The Δ*trpE* mutant showed a significantly smaller hemolytic zone of 8.30 ± 0.27 mm (*p* < 0.001), indicating reduced hemolytic activity. The complemented strain partially restored hemolysis, with a zone diameter of 10.05 ± 0.08 mm, although this value was still significantly different from that of SRW-OG1 (*p* < 0.01) (Figure 2G,H).

Together, these phenotypic results demonstrate that *trpE* deletion compromises multiple virulence-associated traits of *A. salmonicida* SRW-OG1, including biofilm formation, motility, chemotaxis, hemolysis.

### 3.3. Loss of trpE Disrupted Flagellar Formation

**Figure 3:**
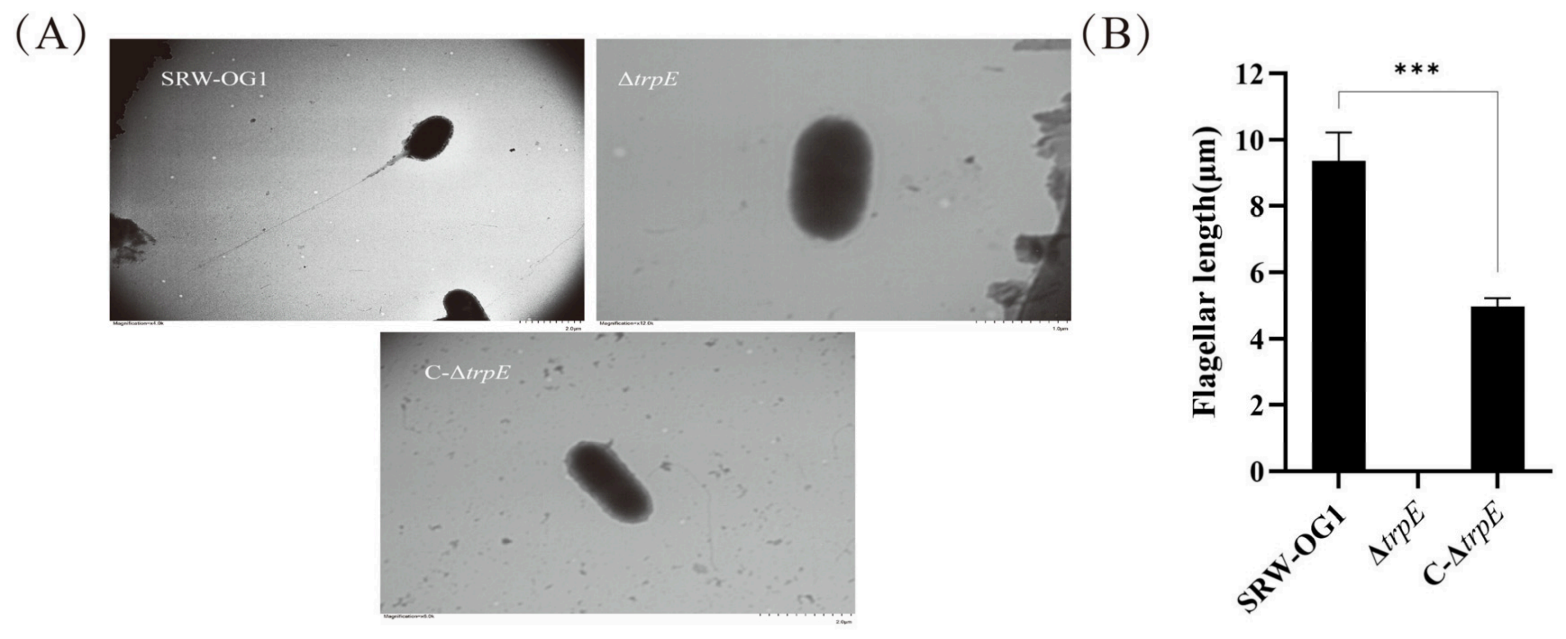
Determination of the flagella of *A. salmonicida* wild-type SRW-OG1, Δ*trpE* mutant strain, and C-Δ*trpE* complementary strain. (**A**) Flagellar morphology; (**B**) flagellar length (μm). *** *p <* 0.001.

Because motility and chemotaxis were both strongly reduced in the Δ*trpE* mutant, transmission electron microscopy was used to examine whether *trpE* deletion affected bacterial and flagellar structure. Morphology of *A. salmonicida* SRW-OG1, Δ*trpE*, and C-Δ*trpE* strains were observated by TEM. As shown in Figure 3, the wild-type SRW-OG1 strain displayed a typical short rod-shaped morphology and possessed visible flagella, with an average flagellar length of 9.13 ± 1.01 μm. In contrast, the Δ*trpE* mutant was completely non-flagellated under the observation conditions. Flagellar formation was partially restored in the complemented strain C-Δ*trpE*; however, the average flagellar length was 4.96 ± 0.26 μm, which was still significantly shorter than that of SRW-OG1 (*p* < 0.001). Flagellar length was quantified by measuring 50 individual bacterial cells for each strain to calculate the average value. These observations indicate that *trpE* is required for normal flagellar formation in SRW-OG1. The loss of flagella in the Δ*trpE* mutant provides a structural-level explanation for the reduced swimming motility and chemotactic ability observed in the phenotypic assays.

### 3.4. Extracellular Product Detection

**Figure 4:**
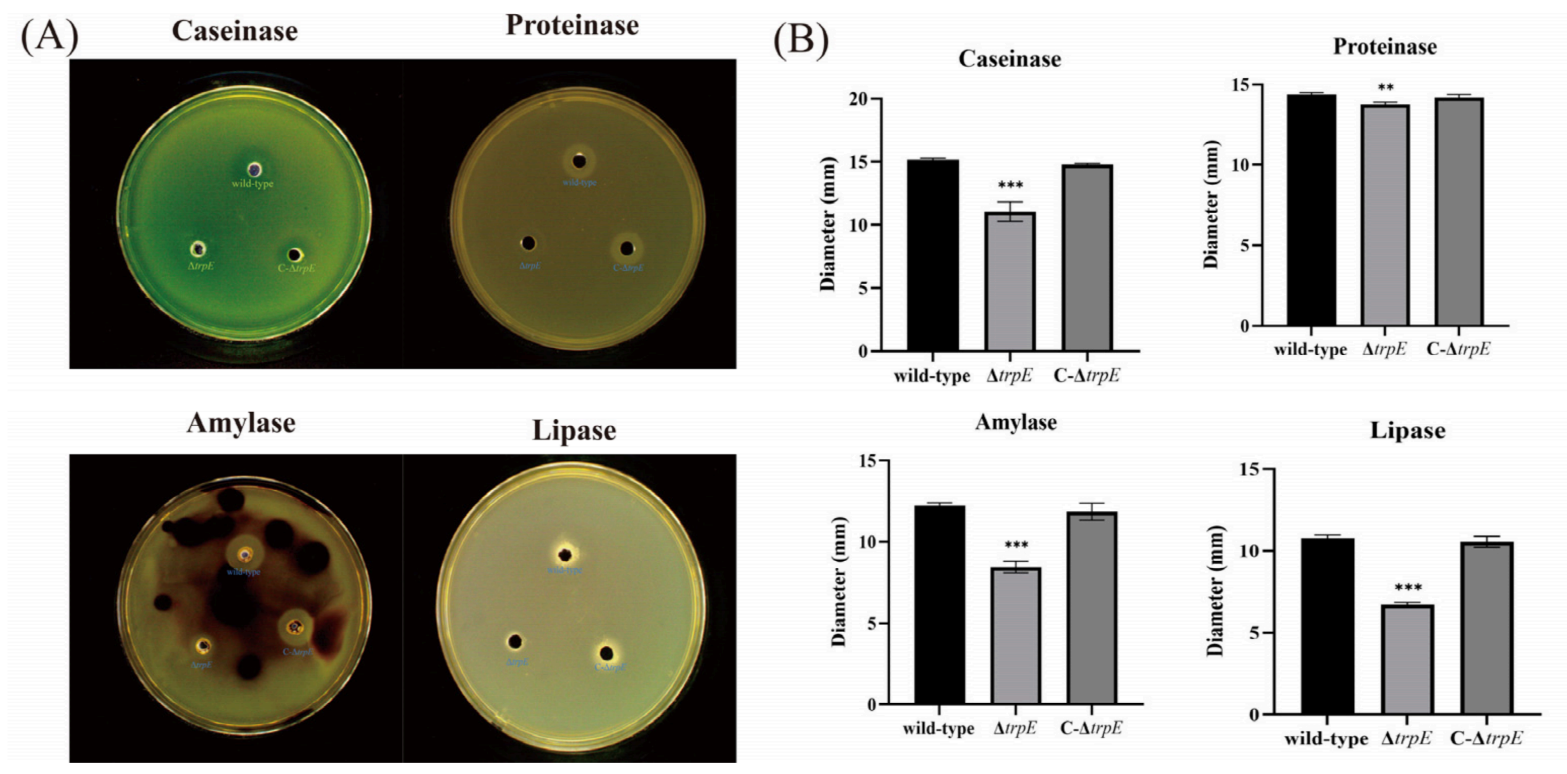
Detection of extracellular product enzyme activities of WT, Δ*trpE*, and C-Δ*trpE*. (**A**) The transparent circles of extracellular product enzyme activity on the plate; (**B**) quantification of hydrolysis zone diameters, ** *p <* 0.01, *** *p <* 0.001.

Extracellular enzyme activities (protease, caseinase, lipase, amylase) were also influenced by *trpE* deletion. They were determined via agar well-diffusion assay, by measuring the diameter of hydrolysis transparent zones formed around the punched wells loaded with bacterial cell-free supernatants. As shown in Figure 4, the wild-type SRW-OG1 strain produced hydrolytic zones of 14.46 ± 0.24 mm for protease, 15.16 ± 0.10 mm for caseinase, 10.96 ± 0.25 mm for lipase, and 12.30 ± 0.28 mm for amylase (Figure 4). In the Δ*trpE* mutant, the corresponding values decreased to 13.78 ± 0.12 mm for protease, 8.30 ± 0.27 mm for caseinase (27.1% decrease, *p* < 0.001), 6.72 ± 0.14 mm for lipase, and 8.45 ± 0.35 mm for amylase, and all reductions were statistically significant (*p* < 0.001). Most extracellular enzyme activities were restored in the complemented strain C-Δ*trpE*, with hydrolytic zone diameters of 14.21 ± 0.16 mm for protease, 14.77 ± 0.11 mm for caseinase, 10.55 ± 0.34 mm for lipase, and 11.86 ± 0.53 mm for amylase, showing no significant differences from SRW-OG1 (*p* > 0.05).

### 3.5. *Δ*trpE Mutant Showed Attenuated Pathogenicity and Partial Protective Efficacy in E. coioides

**Figure 5:**
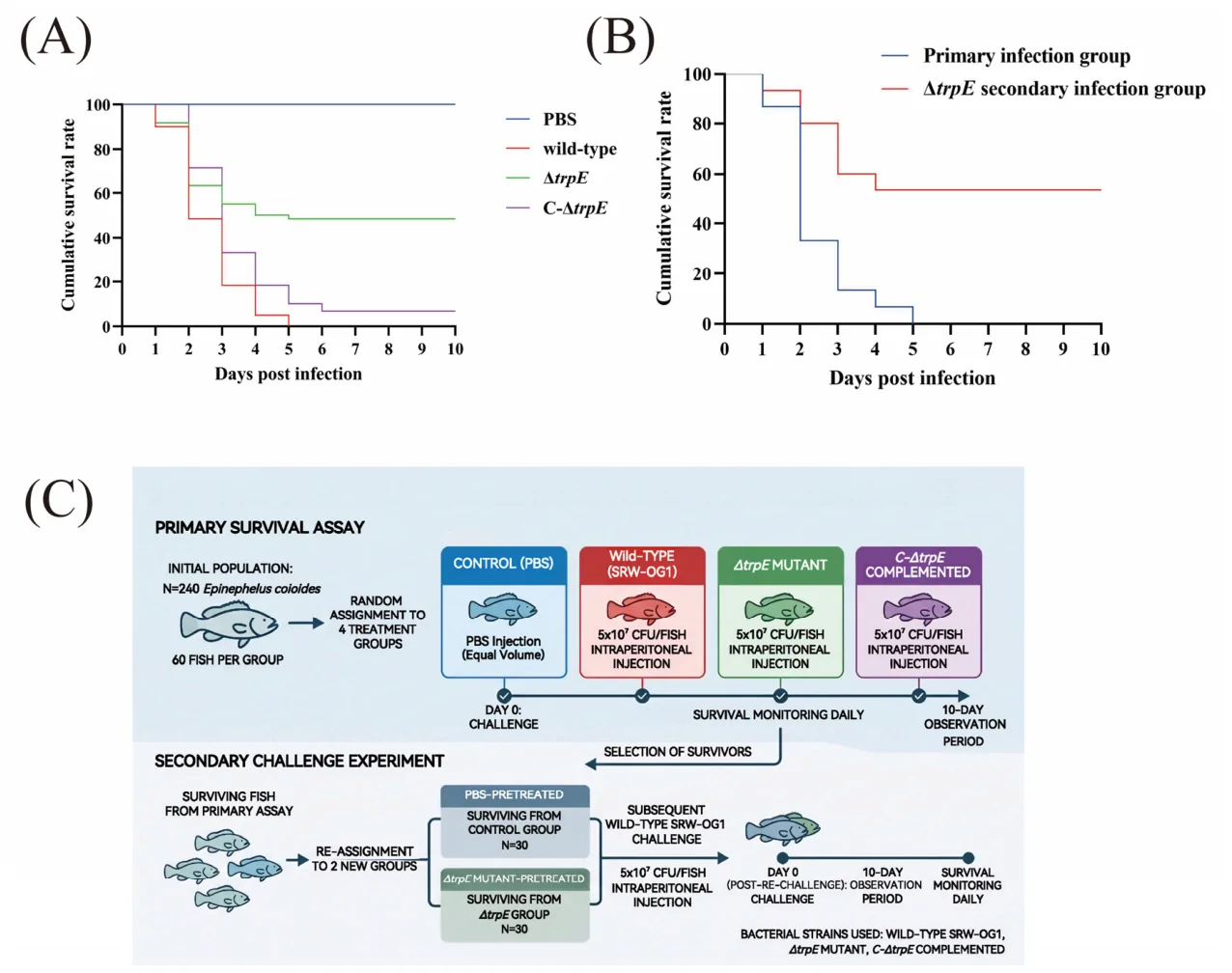
Survival of *E. coioides* following bacterial injected with *A. salmonicida* within 10 days. (**A**) Infection group of Δ*trpE* mutant strain; (**B**) survival rate of fish rechallenged with WT SRW-OG1 after initial pretreatment; (**C**) overall experimental design schematic diagram.

To evaluate the contribution of *trpE* to in vivo pathogenicity, artificial infection experiments were performed in the orange-spotted grouper. The 10-day cumulative survival rate of *E. coioides* injected with *A. salmonicida* is shown in Figure 5A. No mortality was observed in the PBS-injected control group, but fish infected with wild-type SRW-OG1 began to die on the first day post-infection (dpi), and the cumulative mortality reached 100% by 5 dpi. Compared with the wild-type strain, the Δ*trpE* mutant showed markedly attenuated virulence, with a cumulative survival rate of 48.3%. The C-Δ*trpE* infection group showed a cumulative survival rate of 6.7%, indicating that complementation largely restored the virulence of the mutant strain.

The potential protective effect of the Δ*trpE* mutant was further assessed a secondary challenge with wild-type SRW-OG1, the PBS-pretreated group showed 100% mortality by 5 dpi. In contrast, the Δ*trpE* mutant-pretreated group (fish pretreated with Δ*trpE*) showed a survival rate of 53.3% after the secondary challenge (Figure 5B). This suggests that the attenuated Δ*trpE* mutant partial immune protection against subsequent infection with highly virulent SRW-OG1. The overall experimental design is shown in Figure 5C.

**Figure 6:**
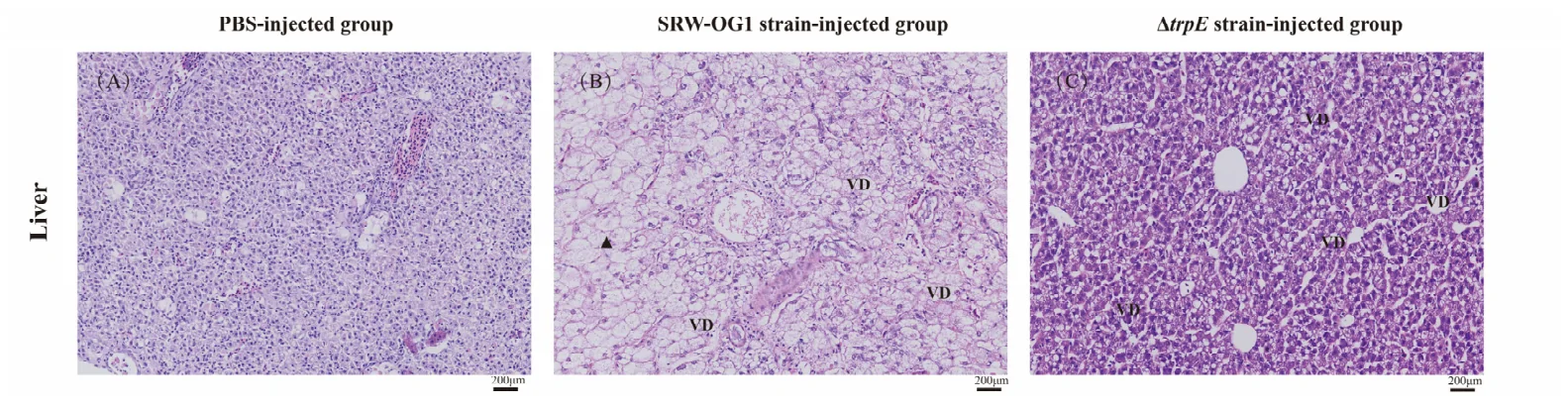
Pathological changes of liver. Liver sections from fish treated with (**A**) PBS, (**B**) WT SRW-OG1, or (**C**) Δ*trpE*. Tissue vacuolation (VD), loss of nuclei and eccentric nuclei (▲).

**Figure 7:**
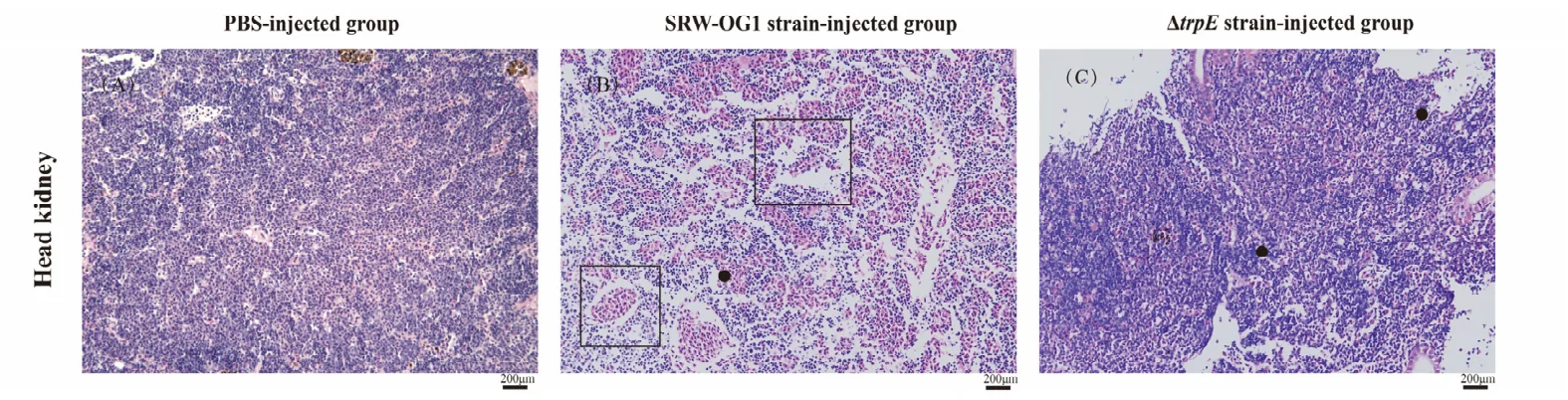
Pathological changes of head kidney. Head kidney sections from fish treated with (**A**) PBS, (**B**) WT SRW-OG1, and (**C**) Δ*trpE*. Dispersed cell arrangement (□), hemagglutination, and cell necrosis (●).

Histopathological examination supported the survival results. As shown in Figure 6 and Figure 7, all images were captured at 200× magnification. Liver and head kidney tissues from the PBS control group showed normal tissue organization, with closely arranged liver cells and compact head kidney cells. In the SRW-OG1 infection group, severe pathological lesions were found, including disorganized liver cell arrangement, nuclear loss, eccentric nuclei, increased vacuolization, hemocyte aggregation, and cell necrosis in the head kidney. Comparatively, the Δ*trpE* mutant exhibited only mild liver vacuolization and slight cell swelling, while hemocyte aggregation and tissue necrosis in the head kidney were substantially alleviated.

**Figure 8:**
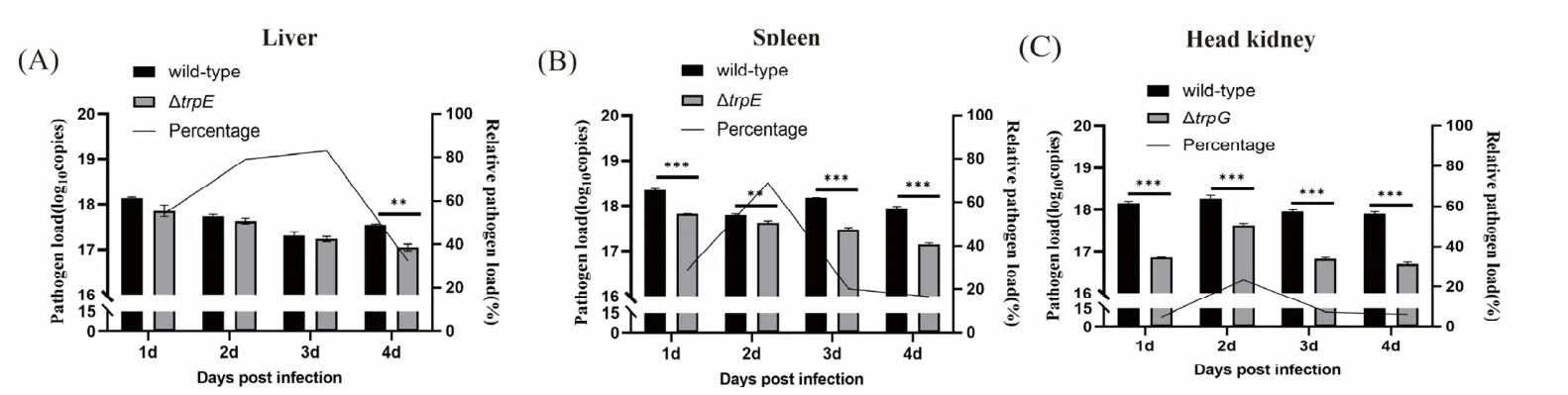
Effects of *trpE* genes on the bacterial loads of WT and Δ*trpE* in the liver, spleen, and head kidney of *E. coioides.* (**A**) The effect of *trpE* on the bacterial load in the liver of *E. coioides*; (**B**) the effect of *trpE* on the bacterial load in the spleen of *E. coioides*; (**C**) the contribution of *trpE* to bacterial load in the head kidney of infected *E. coioides* was determined, ** *p* < 0.01, *** *p* < 0.001.

Bacterial load analysis further confirmed the reduced colonization ability of the Δ*trpE* mutant in host tissues. Bacterial loads in the liver, spleen, and head kidney of *E. coioide*s infected with SRW-OG1 or Δ*trpE* strains were determined over 1–4 days post infection (dpi). In the liver tissue, significantly lower bacterial loads were only detected in the Δ*trpE* mutant group at 4 dpi; throughout the 1–4 dpi time course, bacterial burdens of the Δ*trpE* strain were generally inferior to the wild-type strain in the spleen and head kidney, with statistically significant differences observed at multiple time points across these two immune organs (Figure 8). In SRW-OG1-infected fish, the bacterial load in the liver and head kidney first increased, then decreased, while in the spleen, it showed a "decrease–increase–decrease". The Δ*trpE* mutant maintained a lower bacterial burden throughout the observation period, while the relative bacterial loads of the SRW-OG1 and Δ*trpE* strains in the liver, spleen, and head kidney bottomed out on 4 dpi. This suggests that the deletion of *trpE* reduces the ability of SRW-OG1 to colonize host immune-related tissues and cause tissue damage.

### 3.6. Transcriptomics Reveal Changes in Motility-, Chemotaxis-, and Quorum-Sensing-Related Pathways

**Figure 9:**
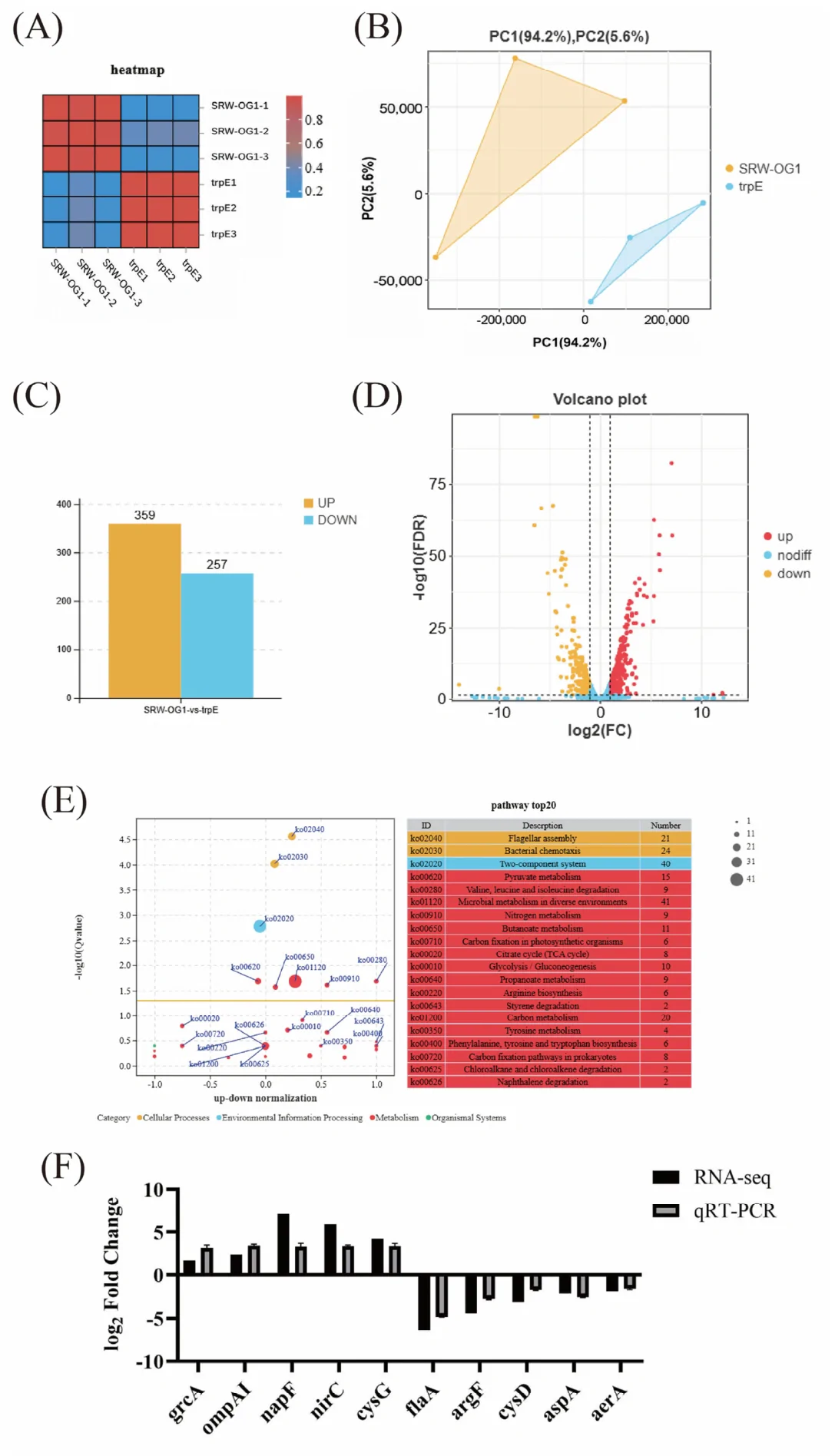
Transcriptomic differences between WT SRW-OG1 and the Δ*trpE* mutant. (**A**) Correlation heatmap between samples; (**B**) PCA diagram between samples; (**C**) histogram of the number of differentially expressed genes, with yellow and blue indicating increased and decreased transcript levels, respectively; (**D**) volcano map of differentially expressed genes; red, blue, and yellow represent up-regulated, unchanged, and down-regulated genes, respectively; (**E**) KEGG enrichment analysis of the top 20 affected pathways. (**F**) qRT-PCR validation of selected differentially expressed genes.

Transcriptomic analysis was performed to compare global gene expression patterns between SRW-OG1 and Δ*trpE* to explore the molecular basis underlying the attenuated phenotypes of the Δ*trpE* mutant. The principal component analysis’s PC1 and PC2 explained 94.2% and 5.6% of the total variance, respectively (Figure 9A,B). Samples within the same group clustered closely, and SRW-OG1 and Δ*trpE* samples were clearly separated, indicating high reproducibility among biological replicates and distinct transcriptional profiles between the two strains.

Using the screening thresholds of FDR and |log2FC| > 1, a total of 616 DEGs were identified in Δ*trpE* relative to SRW-OG1, including 359 upregulated genes and 257 downregulated genes (Figure 9C,D). KEGG enrichment analysis identified 84 enriched pathways, with several closely associated with bacterial virulence, including flagellar assembly, bacterial chemotaxis, two-component systems, quorum sensing, and biofilm formation. Flagellar assembly showed the strongest enrichment, whereas the two-component system contained the largest number of DEGs (Figure 9E). Additionally, the top 20 enriched pathways included pyruvate metabolism and the biosynthesis of phenylalanine, tyrosine, and tryptophan. 

The expression trends were consistent between qRT-PCR (five randomly selected up-regulated and five down-regulated genes) and RNA-seq (Figure 9F), supporting the reliability of the transcriptomic analysis. Thus, deletion of *trpE* broadly affects transcriptional pathways related to motility, chemotaxis, quorum sensing, and biofilm formation, underscoring the observed reductions in virulence-associated phenotypes.

### 3.7. Metabolome Analysis of A. salmonicida SRW-OG1 and ΔtrpE

**Figure 10:**
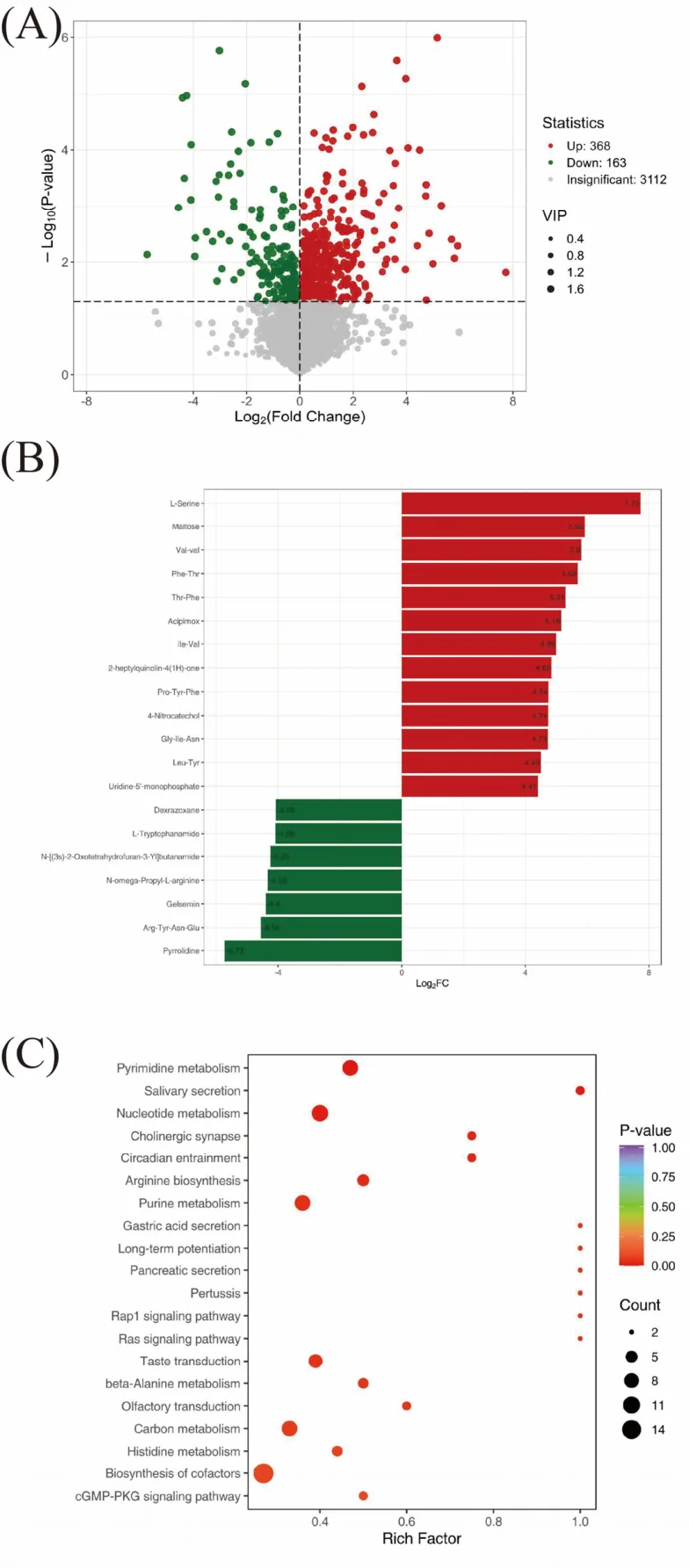
Differential metabolite analysis and KEGG enrichment associated with *trpE* deletion. (**A**) Volcano plot displaying differential metabolites. (**B**) Ranking of the top 20 altered metabolites. (**C**) KEGG enrichment bubble plot showing the top 20 pathways linked to the differential metabolites.

The LC-MS-based untargeted metabolomic analysis using culture supernatants from SRW-OG1 and Δ*trpE* and the *p* < 0.05 and VIP > 1 criteria revealed a total of 531 differentially abundant metabolites. These included 368 upregulated and 163 downregulated metabolites in the Δ*trpE* mutant compared with the wild-type SRW-OG1 (Figure 10A).

Compared with the wild-type SRW-OG1, the top 20 differentially abundant metabolites in the Δ*trpE* mutant are shown in Figure 10B and include: L-serine, maltose, valine-valine, 2-heptyl-4(1H)-quinolone, 4-nitrocatechol, and 5-uridine monophosphate. In contrast, several metabolites were markedly downregulated in the Δ*trpE* mutant, including pyrrolidine, N-ω-propyl-L-arginine, L-tryptophan amide, N-butyryl-L-homoserine lactone (C4-HSL), gelatin, and dexrazoxane. Notably, N-butyryl-L-homoserine lactone is an AHL-type quorum-sensing signal molecule, and the significant reduction in C4-HSL only reflects altered extracellular abundance of this AHL signal metabolite at the metabolic level; this metabolite-level change alone cannot directly confirm impaired in vivo quorum-sensing activity. Combined with our preceding transcriptomic results showing differential expression of dozens of quorum-sensing and two-component system genes, these multi-omics data together imply a potential correlation between *trpE* deletion and disturbed quorum-sensing signal metabolism.

Pyrimidine metabolism, salivary secretion, and nucleotide metabolism were among the most significantly enriched pathways (Figure 10C). Nucleotide metabolism contained 10, pyrimidine metabolism nine, and salivary secretion three enriched metabolites. Cholinergic synapse and circadian rhythm synchronization pathways were also enriched, each containing three metabolites. In addition, cofactor biosynthesis included the largest number of enriched metabolites, with 16 metabolites involved.

This metabolomic data indicate that *trpE* deletion causes broad extracellular metabolic changes and reduces the abundance of an AHL quorum-sensing signal molecule, linking *trpE* to quorum-sensing-associated virulence regulation in *A. salmonicida* SRW-OG1.

### 3.8. Integrated Transcriptome–Metabolome Analysis

**Figure 11:**
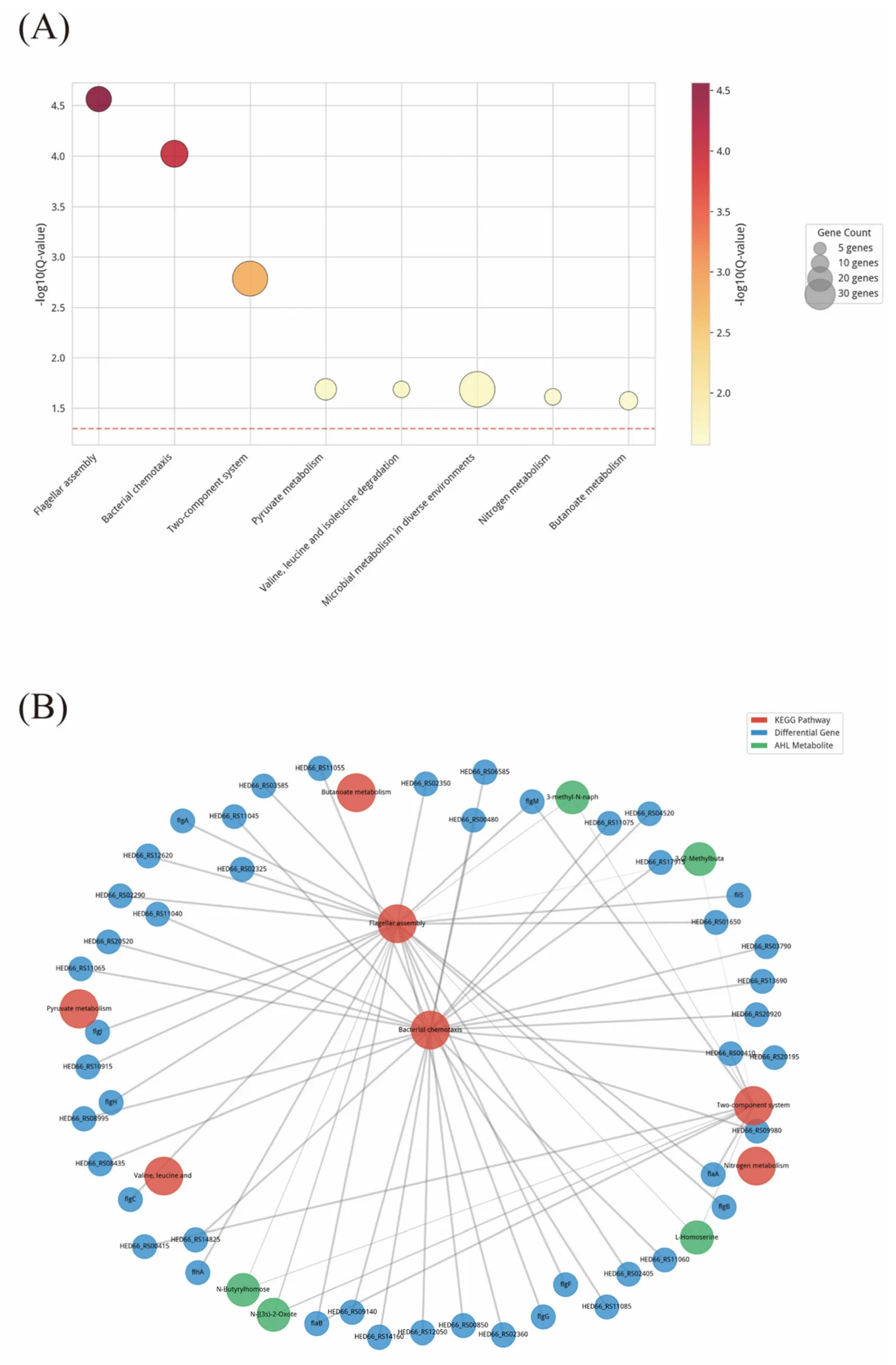
Integrated transcriptomic and metabolomic analysis. (**A**) KEGG pathway enrichment of DEGs (Δ*trpE* mutant vs. wild-type SRW-OG1); (**B**) integrated gene–metabolite–pathway network (Δ*trpE* mutant vs. wild-type SRW-OG1). This figure was preliminarily rendered using Doubao AI (https://www.doubao.com) solely for visualization purposes, with all elements manually verified against source data.

Weighted joint KEGG pathway enrichment analysis and targeted gene–metabolite Pearson correlation network analysis are shown in Figure 11. Joint enrichment identified flagellar assembly and bacterial chemotaxis as the top significantly co-enriched pathways accompanied by C4-AHL reduction, while no flagellar damage-related pathways were statistically enriched, ruling out the reverse feedback model that impaired flagella repress quorum sensing. The correlation network displayed strong positive correlations between C4-AHL and key motility genes *fliA/flhDC* as well as LuxR homologs, without any negative correlation signals supporting reciprocal regulation. The integrated data verify a unidirectional cascade: *trpE* deletion disrupts tryptophan metabolism, limits SAM precursors and lowers C4-AHL abundance; inactivated LuxR cannot trigger transcription of motility genes, and suppressed two-component signals further strengthen this inhibitory effect.

## 4. Discussion

Tryptophan biosynthesis is increasingly connected with microbial pathogenicity, and in addition to its role in amino acid production, it influences bacterial fitness, stress adaptation, host colonization, and the expression of virulence-related traits. Tryptophan-derived metabolites, such as indole and kynurenine-related compounds, have also been implicated in host–pathogen interactions and immune modulation. Therefore, genes involved in the tryptophan biosynthetic pathway may function as important metabolic regulators of bacterial virulence. Specifically, *trpE* encodes the α-subunit of anthranilate synthase, a key enzyme that catalyzes the conversion of chorismate to anthranilate, the first committed step in tryptophan biosynthesis [21]. In *Thermococcus kodakarensis*, disruption of *trpE* abolishes autonomous tryptophan synthesis and produces a tryptophan–auxotrophic phenotype [22]. In this study, LC-MS-based untargeted metabolomic analysis showed that deletion of *trpE* in *A. salmonicida* SRW-OG1 significantly affected the tryptophan biosynthetic pathway, as reflected by the marked decrease in L-tryptophan amide in the Δ*trpE* mutant. This observation aligns with prior data from *Ralstonia pseudosolanacearum* and indicates a conserved correlative relationship between intact *trpE* and steady tryptophan metabolic homeostasis [9].

A disrupted *trpE* gene reduces the ability of bacteria to synthesize tryptophan, resulting in a tryptophan deficiency, highlighting the indispensable role of *trpE* in *A. salmonicida* and its importance in bacterial communication and community-level regulation. For example, blocking this pathway with glyphosate reduces the virulence of *Cryptococcus neoformans* [23], and disruption of tryptophan synthesis in *Histoplasma capsulatum* [24] restricts intracellular proliferation in macrophages while attenuating virulence. Similarly, mutation of *trpE* in *Ralstonia pseudosolanacearum* markedly decreases the expression of type III secretion system-related genes [9], while deletion of *trpE* in *Salmonella enterica* reduces bacterial adhesion and biofilm formation. Notably, this biofilm defect can be rescued by exogenous tryptophan or indole [13]. Reduced infectivity has also been reported in *Aspergillus fumigatus* Δ*trpE* mutants [14], suggesting that the contribution of *trpE* to pathogenicity may be relatively conserved in microorganisms, although downstream phenotypes and regulatory mechanisms may differ. Anthranilate synthase, AS-I (TrpE), is the key enzyme for bacterial tryptophan synthesis, and interfering with tryptophan reduces *Mycobacterium tuberculosis* virulence while knockdown of *trpE* enhances the susceptibility of *M. tuberculosis* to Chuangxinmycin. Additionally, the Δ*trpE* mutants resulted in a decreased affinity for tryptophan [25]. Collectively, these cross-species reports reveal that *trpE* participates in virulence modulation across microbes, yet distinct species exhibit divergent phenotypic outputs and underlying regulatory logic. The function of *trpE* in *A. salmonicida* had not been clearly characterized before, but results showed that the Δ*trpE* mutant exhibited reduced growth, indicating that *trpE* contributes to the physiological fitness of SRW-OG1. Because tryptophan is required for protein synthesis and possibly metabolic homeostasis, impaired growth in the mutant is likely linked to the disruption of tryptophan biosynthesis. This growth defect may contribute to its weakened virulence, but the coordinated reduction in motility, chemotaxis, biofilm formation, hemolytic activity, and extracellular enzyme activity suggests that *trpE* influences pathogenicity through multiple avenues.

Motility is critical for bacterial pathogens, as it allows cells to move toward favorable environments, approach host tissues, and initiate colonization. This can be done through swimming, swarming, twitching, gliding, and sliding motility, each of which can contribute to environmental adaptation and host interaction [26]. Also, planktonic and biofilm modes play distinct roles in bacterial propagation and colonization. Current data show that the growth, swimming motility, and hemolytic activity of the Δ*trpE* strain were significantly lowered (*p* < 0.001), consistent with results in *Salmonella typhimurium* and *Pseudomonas aeruginosa* [13], highlighting the gene’s importance in facilitating bacterial pathogenic biological characteristics. This aligns with accumulating evidence that *trpE* plays an important regulatory role in bacterial pathogenicity across different species. 

Additionally, deletion of *trpE* significantly reduced swimming motility and chemotactic ability in SRW-OG1, due to the Δ*trpE* mutant being completely non-flagellated. Notably, flagellar formation was partially restored in the complemented strain, with flagellar length reduced by −53% compared to SRW-OG1’s 9.13 μm (*p <* 0.001). These correlative observations imply *trpE* participates in flagellar biogenesis or maintenance in *A. salmonicida*; the full loss of flagella only provides a plausible structural basis for the mutant’s defective motility and chemotaxis. Similar links between tryptophan biosynthesis and motility phenotypes have been reported in other bacteria [27], supporting the idea that amino acid metabolism can affect cellular structures required for colonization and infection.

Biofilms support bacterial survival under environmental stress, facilitate adhesion to host surfaces, and promote persistent infection. In the Δ*trpE* mutant, biofilm biomass was markedly lower than in the wild-type strain, whereas complementation partially restored this phenotype. Similarly, *trpE* deletion reduces biofilm formation in *Salmonella Typhimurium* and *Escherichia coli* [13]. In *Salmonella enterica* serovar Typhimurium, the biofilm defect of the Δ*trpE* mutant is associated with reduced cellulose production, reversible by supplementation with tryptophan or indole [13]. In *E. coli*, intracellular tryptophan levels may also influence biofilm development [11], so that disruption of tryptophan biosynthesis may weaken bacterial biofilm formation by altering metabolic status and downstream regulatory pathways.

In addition, the Δ*trpE* strain showed significant reductions in hemolytic activity and the extracellular activities of caseinase, lipase, protease, and amylase, compared with the SRW-OG1 strain. Extracellular enzymes affect virulence in aquatic pathogens because they contribute to nutrient acquisition, tissue invasion, and host damage. For example, extracellular protease activity has been associated with the pathogenicity of *Vibrio parahaemolyticus* [28]. The observed decline in extracellular enzyme activities in the Δ*trpE* mutant may therefore reflect a reduced ability to damage host tissues or exploit host-derived nutrients during infection. In *Xanthomonas oryzae* pv. *oryzae*, *trpE* knockout also results in attenuated virulence, possibly due to tryptophan auxotrophy [29]. So, the reduced extracellular enzymatic activities and the loss of flagella in SRW-OG1 may be downstream consequences of altered metabolic homeostasis caused by *trpE* deletion. These results broaden the functional relevance of *trpE* from amino acid biosynthesis to multiple pathogenicity-related processes.

Further corroborating the in vitro, in vivo artificial infection experiments with *E. coioides* provided direct and partial survival-based evidence for its essential function in the pathogenicity of *A. salmonicida* SRW-OG1. Fish infected with the Δ*trpE* mutant showed a cumulative survival rate of 48.3%, whereas wild-type SRW-OG1 caused 100% mortality by five days post-infection. The C-Δ*trpE* strain largely restored virulence, indicating that the reduction in pathogenicity was primarily due to the deletion of *trpE*. The Δ*trpE* of *Xanthomonas oryzae* also resulted in a significant reduction in the pathogenicity of the host [29], while complementation of *cadA* and *cadB* has not reached strain wild-type levels in motility, biofilm formation, and pathogenicity [12]. Histopathological analysis also confirmed that Δ*trpE* caused substantially milder liver and head kidney lesions than the wild-type strain, where severe tissue damage was characterized by hepatocellular disorganization, vacuolization, hemocyte aggregation, and cell necrosis. These pathological changes were clearly alleviated in the Δ*trpE* infection group, which maintained lower burdens in the liver, spleen, and head kidney throughout the infection period. Taken together, these in vivo results indicate that *trpE* deletion compromises the colonization and pathological capacity of SRW-OG1. This aligns with previous studies showing that tryptophan metabolism-related genes play a conserved regulatory role in bacterial virulence, similar to the *trpA* gene mediating virulence in *Vibrio anguillarum* [10]. This also underscores the universal importance of tryptophan synthesis pathways in fish bacterial pathogens.

Notably, pretreatment with Δ*trpE* conferred 53.3% protection against subsequent challenge with the virulent wild-type strain, whereas PBS-pretreated fish showed complete mortality. This suggests that the Δ*trpE* mutant has potential as a live attenuated vaccine candidate, retaining sufficient immunogenicity while showing reduced pathogenicity. Similar reductions in host virulence have been observed after *trpE* deletion in *Xanthomonas oryzae* [29], supporting exploration of live attenuated vaccines against *A. salmonicida* [30] and highlighting the practical value of *trpE* deletion in vaccine candidate development.

Transcriptomic analysis revealed a total of 616 differentially expressed genes between SRW-OG1 and Δ*trpE*, including 359 upregulated and 257 downregulated genes. Not surprisingly, genes associated with motility, chemotaxis, and biofilm formation were significantly downregulated, aligning with the phenotypic observations. Gene Ontology (GO) annotations point to *trpE* as a broad influencer of basic cellular functions that support QS-mediated virulence, while KEGG enrichment shows that several affected pathways were directly relevant to the phenotypes observed in this study, including flagellar assembly, bacterial chemotaxis, two-component systems, quorum sensing, and biofilm formation. This supports the absence of flagella and the reduced motility and chemotactic response observed in the mutant. Two-component systems are well-documented to cross-regulate QS in bacteria [31], and their dysregulation in Δ*trpE* suggests a potential regulatory cascade. Here, *trpE* may modulate two-component signaling, which in turn controls QS-mediated expression of flagellar genes (e.g., downregulation of the flagellar filament protein gene *fliC* in Δ*trpE*) and other virulence-associated factors, disrupting QS-coordinated flagellar assembly and function, and ultimately impairing motility and colonization.

A total of 531 differentially abundant metabolites (368 upregulated, 163 downregulated) were detected between the Δ*trpE* mutant and the wild-type strain, with a key QS signal molecule—N-butyryl-L-homoserine lactone (a member of the AHL family)—significantly downregulated in Δ*trpE*. This supports a link between *trpE*, quorum sensing, and virulence regulation. Specifically, N-butyryl-L-homoserine lactone was of particular interest because it belongs to the acyl-homoserine lactone family of quorum-sensing signals [12]. Since AHL-mediated quorum sensing (QS) coordinates bacterial group behaviors, including biofilm formation, motility, extracellular enzyme secretion, and virulence factor production, it reduces C4-HSL [32]. This reduction in extracellular C4-HSL reflects altered metabolite abundance and cannot independently confirm impaired functional QS activity; combined with transcriptional repression of QS and motility pathways, this metabolic shift provides a correlative metabolic correlate for the mutant’s QS-linked phenotypic defects. The decrease in N-butyryl-L-homoserine lactone possibly explains the simultaneous attenuation of several quorum-sensing-associated phenotypes in Δ*trpE*. Although metabolic changes, such as the increase in L-serine, may contribute to impaired growth or altered cellular physiology, the reduction in an AHL signal is especially relevant to the observed defects in biofilm formation, motility, and extracellular enzyme activity.

Current results thus propose that *trpE* contributes to the virulence of *A. salmonicida* SRW-OG1 through a metabolism-associated regulatory mechanism. Briefly, disruption of *trpE* interferes with tryptophan biosynthesis and changes the extracellular metabolic profile, including the reduction in L-tryptophan amide and an AHL quorum-sensing signal. These metabolic changes result in the altered expression of genes involved in flagellar assembly, chemotaxis, quorum sensing, two-component regulation, and biofilm formation and ultimately weakened growth, impaired flagellar formation, reduced motility and chemotaxis, decreased biofilm formation and extracellular enzyme activities, lower tissue colonization, and attenuated virulence in the host. This highlights a possible connection between tryptophan metabolism and quorum-sensing-mediated pathogenicity in *A. salmonicida*.

## 5. Conclusions

This study demonstrates that *trpE* plays an important role in the regulation of the virulence of mesophilic *A. salmonicida* SRW-OG1. Deletion of *trpE* disrupted tryptophan-related metabolism, impaired growth and multiple virulence-associated phenotypes, reduced host tissue colonization, and attenuated pathogenicity in *E. coioides*. The reduction in an AHL quorum-sensing signal and the transcriptomic changes in motility- and biofilm-related pathways suggest that *trpE* connects tryptophan biosynthesis with quorum-sensing-associated virulence regulation. The attenuated phenotype and partial protective effect of Δ*trpE* highlight its potential as a live attenuated vaccine candidate for the prevention of *A. salmonicida* infection in aquaculture.

## Figures and Tables

**Table 1:** Primer sequence of *trpE*.

Name	Sequence (5′–3′)	Expected Amplicon Size (bp)	GenBank Accession No.
*trpE*	F: ATACGACTCGCTTGCCATGAGCCATATTCAACGGGAA R: GTATCCATCTCCCCCGGAAAAACTCATCGAGCATCAA	845	MT525253.1
C-*trpE*	F: CGAGCTCATGACCGTCAATACGAC R: CTCTAGATTAGTCATGGCTCACG	1638	MT525253.1
*trpE*-p	F: AGGCATTTTTTCAAGGTAT R: CGGCAGGCTGTTGCGGTAG	WT: 1893; Δ*trpE*: 1309; C-Δ*trpE*: 1908	MT525253.1

**Table 2:** Primers for qRT-PCR verification.

Name	Sequence (5′–3′)
*gyrB*	F: GGGGTCTACTGCTTCACCAA R: CTTGTCCGGGTTGTACTCGT
*grcA*	F: GTCACAACGTCACGCTGCTG R: GGAAGACGCCATCGCCAATC
*ompAI*	F: ACGGGCACGGGCTTCAG R: GTACCAGCAGATCGTTGACTTCC
*napF*	F: TCATGTTGGAGCCTCGGATCTG R: GCCGACTCCCAGCATAGATACC
*nirC*	F: CGCCAAGTTCATCGCCATCTG R: AATGGGCACCGAACCAGGAG
*cysG*	F: CCGACAGGCGTGAAGTCAATG R: ATGATCGACGGCATGATGAAGC
*flaA*	F: GCCACCACTGAGCAAGTTCTG R: CAACAACACCGATGACCGTACC
*argF*	F: TTCATCCCATATTTGTCAGCCACTTC R: GTCATCAACGCCACCAAGAACC
*cysD*	F: CTACAACAGCCAGGTCAACAAGG R: GCGGCACTATGTCGATGTTCTC
*aspA*	F: GTACTTCGTTGGTGTTCATGTTGAC R: TAGAAGCCTGCGACCTGATGC
*aerA*	F: AACCGCCCGAACTGGAACC R: CCAATCCCACCACTTCACTTCAC

## Data Availability

Raw experimental data are not publicly archived due to laboratory storage regulations but are accessible from the corresponding author on reasonable request.
